# Physicochemical and control releasing properties of date pit (*Phoenix dactylifera* L.) phenolic compounds microencapsulated through fluidized‐bed method

**DOI:** 10.1002/fsn3.3173

**Published:** 2022-12-13

**Authors:** Kasra Afshari, Majid Javanmard Dakheli, Yousef Ramezan, Alireza Bassiri, Hossein Ahmadi Chenarbon

**Affiliations:** ^1^ Department of Food Science and Technology, Faculty of Pharmacy, Tehran Medical Sciences Islamic Azad University Tehran Iran; ^2^ Food Technologies Group, Department of Chemical Technologies Iranian Research Organization for Science and Technology (IROST) Tehran Iran; ^3^ Department of Agronomy, College of Agriculture, Varamin ‐ Pishva Branch Islamic Azad University Varamin Iran

**Keywords:** date pit, fluidized‐bed drying, microencapsulation, phenolic compounds

## Abstract

This study aimed to investigate the effect of different ethanol/water solvents on phenolic compound extraction and microencapsulated extract of date pit powder. The highest and the lowest amounts of total phenolic compounds were 742.37 and 236.07 mg GAE/g dm, respectively, observed in water–ethanol composite solvent (25% W: 75% E) and water solvent (100% W). In this regard, the highest and lowest values of IC_50_ were 6.83 and 0.90 μg/ml, measured in water solvent (100% W) and water–ethanol solvent (25% W: 75% E), respectively. In the second phase, using maltodextrin (10%, 20%, and 30% W/V) as the first layer, date pit extract was microencapsulated. *Alhagi maurorum* gum (10%, 20%, and 30% W/V) as the second layer and medium‐chain triglycerides (MCT oil) (15% W/W) as the third layer were used by a fluidized‐bed drying technique. By increasing temperature, the microencapsulated extract powder solubility was increased as well. In contrast, the moisture content, bulk density, tapped density, and compressibility index decreased. By increasing temperature, the maltodextrin and *A. maurorum* gum concentration, the coating efficiency, and the loading capacity of the samples increased initially and decreased eventually. Moisture content, powder solubility, bulk density, and compressibility index increased, with increasing maltodextrin concentration, however, tapped density decreased. The optimal physicochemical properties of the phenolic compounds’ microcapsules were determined at 45°C and at a concentration of 20% of each of the maltodextrin and *A. maurorum* gum. According to scanning electron images, the powder particles were spherical and had a relatively smooth surface. Notably, the release rate of phenolic compounds reached its maximum (64%) after 24 h.

## Graphical abstract

In this research, the date pit extract was fluidized bed and encapsulated by maltodextrin, Manna of Hedysarum gum, and MCT oil (as a hydrophobic layer). The effect of different parameters on the efficiency and the microencapsulation properties was studied. Resulted, encapsulated phenolic compounds can be used as functional food ingredients.

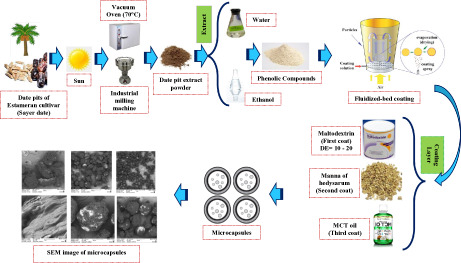

## 1 INTRODUCTION

Date trees (*Phoenix dactylifera* L.) are widely cultivated in arid and semiarid environments. They are considered an important crop in North Africa and the Middle Eastern region (Chao & Krueger, 2007). The annual world production of dates is around 9,075,446 tons (FAOSTAT, 2019). Depending on the variety, date pits represent 6.10%–11.4% of the total weight of the fruit (Ourradi et al., 2021); they contain a high percentage of carbohydrates (81.0%–83.1%), protein (5.17%–5.56%), oil (10.19%–12.67%), ash (1.12%–1.15%), and oleic acid (41.3%–47.7%) (Mohammadi et al., 2016). Date pits also contain substantial amounts of secondary metabolites, mainly phenolic compounds (3102–4430 mg gallic acid equivalent /100 g), antioxidants (580–929 μm Trolox equivalent/g), and dietary fiber (78–80 g/100 g). Phenolic compounds have been shown to possess such benefits as being antioxidant, anticarcinogenic, antimicrobial, and anti‐inflammatory (Baliga et al., 2011; Habib & Ibrahim, 2009). The industrial processing of this fruit results in the rejection of considerable quantities of waste, represented mainly by the pits (Messaoudi et al., 2013). Many practices have been established already to valorize this agroindustrial by‐product, mainly as a precursor to producing activated carbon or used in animal feed. Antioxidant activity is closely related to the phenolic content of plants (Awulachew, 2021; Oladzad et al., 2021). In recent years, great interest has been focused on using natural antioxidants in food products since the studies have indicated that possible adverse effects may have been related to the consumption of synthetic antioxidants (Hasheminya & Dehghannya, 2020; Lourenço et al., 2019; Negi, 2012). Phenolic compounds are unstable and sensitive to high temperature, light, pH, and oxidative and degradative enzymes, which affect the phenolic profile of extracts. Therefore, finding a strategy to protect phenolic compounds and preserve their biological activities and properties seems vital. Encapsulation is a common technique for creating an external membrane or coating of one material over another material and provides both stabilization and a controlled release of the entrapped materials, resulting in the conversion of volatile liquid into stable solid‐encapsulated products, protection of the active compounds against environmental factors (such as oxygen, light, moisture, and pH), reduction in flammability, and increasing water dispersibility, thereby, improving handling ease, safety, and applicability to various products.

The coating enables the controlled release of compounds at the targeted site and specific rate (Budinčić et al., 2021; Li et al., 2020; Martínez‐Ballesta et al., 2018). In the case of antioxidants, the active agent should be released slowly and continuously at an optimal threshold in the food by encapsulation.

The release kinetics of the active elements within the microcapsules depends directly on the processes and formulation parameters which are specifically designed to release components when subjected to certain parameters (Hameed et al., 2020). The active ingredient can be released through two methods; forced and controlled release. The forced release is obtained by rupturing the microcapsule membrane under thermal and/or mechanical conditions, such as friction. The controlled release is based on the diffusion of the encapsulated active element through the membrane or its degradation. Various processes, such as extrusion, coacervation, cocrystallization, lyophilization, spray drying, and fluidized‐bed coating, are employed to encapsulate and coat food ingredients or additives, in fluidized‐bed encapsulation, while the core material particles are suspended, the wall material is atomized into the chamber, depositing on the core. When the particles reach the top of the ascending column, they are released into a descending column of air that releases them back into the fluidized bed, where they are again coated, dried, and hardened, ensuring a uniform coating. The simplest encapsulation technique consists in making soluble ingredients in a solution containing the wall material, followed by spray drying. There are many published works on spray‐drying encapsulation of phenolic extracts from plant sources, such as pomegranate peel (Šavikin et al., 2021), berries (Etzbach et al., 2020), bay leaves (Medina‐Torres et al., 2016), carrots (Wagner & Warthesen, 1995), olive leaves (Dobrinčić et al., 2020), sour cherry pomace (Başyiğit et al., 2020), soybean (Poomkokrak et al., 2015), and date pit oil (El‐Massry et al., 2019).

According to studies, no attention has been paid to date fruit wastes, such as pit, which is a rich source of phenolic compounds. Microencapsulation of extracts containing phenolic compounds, as a natural antioxidant, is one of the approaches to prevent oxidation and extend its shelf life.

This study aimed to investigate microencapsulation and controlled release of phenolic compounds extracted from date pit produced by the fluidized‐bed method. It should be noted that using the fluidized‐bed method for encapsulation and other ingredients, such as MCT oil, *Alhagi maurorum* gum, and maltodextrin in three layered coating on phenolic compounds, are innovative aspects of this study.

## 2 MATERIALS AND METHODS

### 2.1 Materials

Date pits of Estameran cultivar (Sayer date) were purchased from Parsian Date Chocolate Company (Ahvaz city, Khuzestan province, Iran). Maltodextrin (DE = 10 and 20), *A. maurorum* gum, medium‐chain triglycerides (MCT), microcrystalline cellulose (Avicel ph‐20), chemicals, and solvents were purchased from Sigma corporation.

### 2.2 Sample preparation

Date pits were cleaned and washed with distilled water. After washing, they were dried in a vacuum oven (Memmert VO400, Germany) at 70°C until the moisture content reached 5%, and by using an industrial milling machine (Model S‐G5 500 Swantek, made in Germany) were milled. The powder was passed through a sieve with a diameter of 710 microns (mesh 25) and stored in polyethylene bags at 4 ± 2°C.

### 2.3 Extraction of phenolic compounds

Water (100%) and ethanol (100%), as well as water–ethanol (50/50, 35/65, 25/75 v/v), were used as extraction solvents due to Moradi et al. (2022) method with some modifications. Ten gram of date pit powder was poured into Erlenmeyer containing 100 ml of the desired solvents and stirred for 5 h at 25°C in an incubator with a shaker (PIT053RS, Iran) at 280 rpm. Then, the suspension was filtered by Büchner funnel and Whatman filter paper (≠4). All samples were kept in the refrigerator at 4°C until the tests were performed.

### 2.4 Measurement of total phenolic compounds

The total phenolic compounds were measured using the colorimetric method and the Folin–Ciocalteu reagent. One milliliter of the extract was mixed with 2.5 ml of 10% folic acid reagent diluted in water. After 8 min, 5 ml of 7.5% sodium carbonate was added, and then the volume of the solution was increased to 50 ml with distilled water. The resulting mixture was kept in the dark condition for 30 min, and finally, the absorbance of the samples was measured using spectrophotometry at 765 nm. The total amount of phenolic compounds was stated as mg/g dry weight of the extract using the line equation drawn for Gallic acid (Selahvarzi et al., 2022).

### 2.5 DPPH free radical scavenging

Antioxidant activity (%) was evaluated by DPPH free radical assay. The DPPH radical scavenging activity was measured according to the methodology described by Kashfi et al. (2020). One milligram of date pit extract, as well as TBHQ synthetic antioxidant compounds, was prepared in pure ethanol solvent. Then, 0.3 ml of the prepared solution was mixed with 2.7 ml of pure ethanol solution containing DPPH reagent (concentration 0.1 mM). The stirred mixture was kept in the dark for 60 min. Subsequently, the adsorption was measured at 517 nm using a spectrophotometer (Bruker Optik, Ettlingen, Germany), and the percentage of the radical reduction in DPPH free radicals was calculated using Equation (1) (Kashfi et al., 2015).

(1) $$\text{Inhibition}\;\left(\%\right)=\frac{A_{o}-A_{s}}{A_{o}}\times 100$$

where *A*_*o*_ = sample absorption at time zero and *A*_*s*_ = sample absorption after 60 min.

### 2.6 Microencapsulation of the extract by fluidized‐bed drying method

**FIGURE 1:**
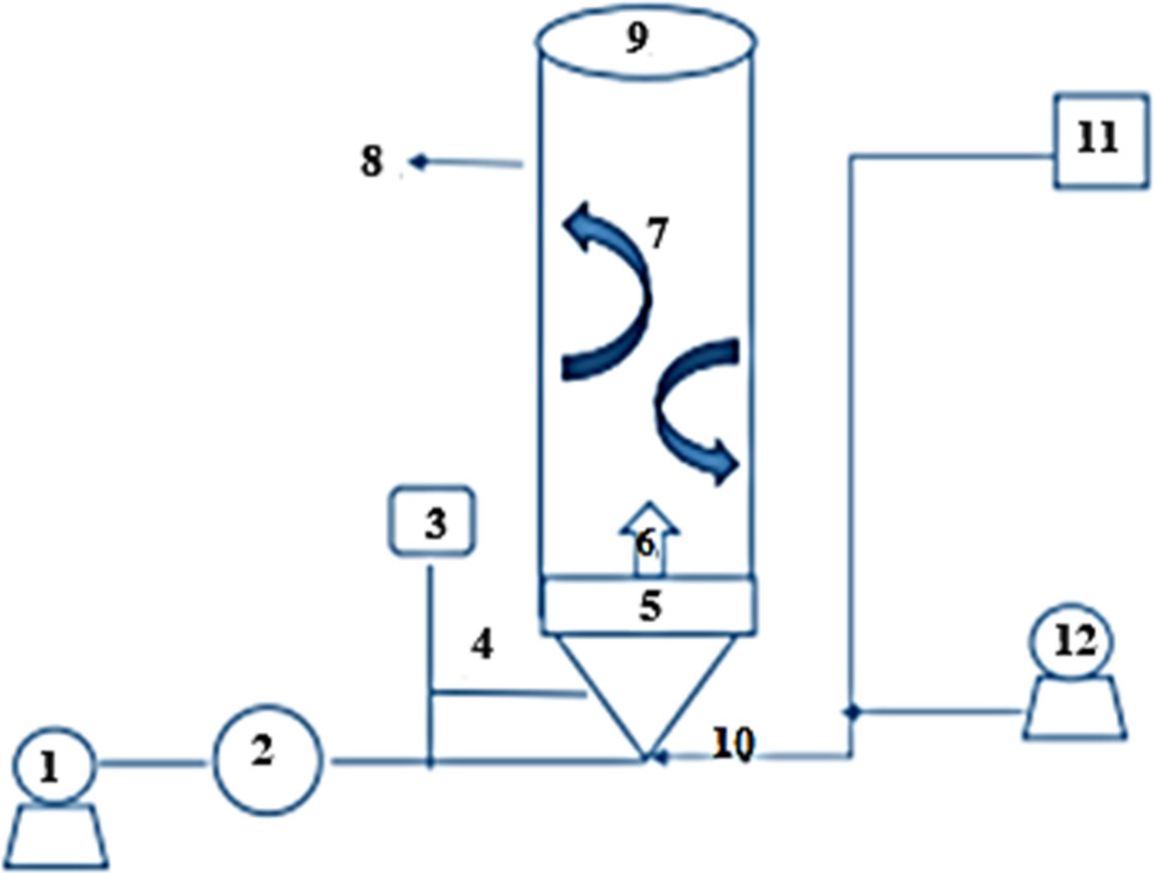
Schematic diagram of pilot‐scale fluidized‐bed dryer: (1) pressure pump, (2) pressure gauge, (3) inlet of coated solution, (4) flow control valve, (5) inlet air distribution plate and filter, (6) nozzle, (7) particle movement path, (8) middle tube of device, (9) inlet of carrier powder, (10) inlet airflow, (11) digital thermometer, and (12) air pump.

The extracts were coated using maltodextrin (first layer), *A. maurorum* gum (second layer), and MCT oil (third layer) in the temperature range (45, 50, 55, and 60°C) by a fluidized‐bed dryer (Figure 1). Five gram of microcrystalline cellulose (MCC) powder (Avicel ph‐20), as core and neutral carrier, was poured on the bottom of the fluidized bed. The MCC flowed at 0.25 ml/min rate, and air with 300 mbar pressure was used to produce nonagglomerated granules.

Five milliliter solution containing date pit extract was sprayed (airflow at a pressure of 150 mbar) at concentrations of 30%, 40%, and 50% on floated MCC. For the first layer, 5 g of DE = 10, 20, and 30 maltodextrins (a pressure pump of 300 mbar with a flow rate of 0.25 ml/min) were sprayed on the granules (MCC + date pit extract) of the previous stage, which was floating at a pressure of 150 mbar. For the second layer, *A. maurorum* gum solution (°Brix 12) in concentrations of 10%, 20%, and 30% was prepared. The solutions were sterilized in an autoclave at 121°C for 15 min. Coating solutions then were sprayed by a pump at a pressure of 300 mbar with a flow rate of 0.25 ml/min on the granules of the previous stage (MCC + date pit extract + DE), which were floating at 100 mbar. A hydrophobic layer of MCT oil with a 15% concentration was used as the third and outer wall of the capsules to prevent the capsules from rupturing.

### 2.7 Measurement of physicochemical properties of microcapsules

#### 2.7.1 Measurement of the moisture content

The samples were dried in an oven (Memmert UF30, Germany) at 105 ± 2°C for 6 h until reaching a constant weight. Then, the moisture content of the samples was calculated according to Equation (2) (Shahidi & Molaveasi, 2020).

(2) $$M\left(\%\right)=\frac{W_{2}-W_{3}}{W_{2}-W_{1}}\times 100$$

where *M* is the moisture content, *W*_1_ is the weight of the empty container (g), *W*_2_ is the total weight of the powder and the container (g), and *W*_3_ is the total weight of the dried powder and the container (g) after putting in the oven.

#### 2.7.2 Solubility of powder

One gram of the produced powder was poured into 100 ml of distilled water to determine the solubility. The sample was put in a centrifuge (Sigma 2‐16P, Germany) at 112 RCF for 10 min to separate the insoluble parts. The supernatant separated from the centrifuge tube was poured into a fixed‐weight glass container and dried at 105°C until reaching a constant weight. The solubility percentage of the powder was determined from the difference between the weight of primary and secondary dry matter (Goula & Adamopoulos, 2010).

#### 2.7.3 Bulk density measurement

Five gram of the produced powder was poured into a 10 ml graduated cylinder, and the cylinder was shaken slightly to smooth the surface of the powder inside the cylinder. Then, according to Equation (3), the bulk density was obtained from the ratio of powder mass to the volume occupied in the cylinder (Goula & Adamopoulos, 2010).

(3) $$\rho_{b}=\frac{m}{V}$$

where *m* is the mass of the powder (g) and *V* is the sample volume in ml.

#### 2.7.4 Tapped density measurement

After determining the bulk density, continuous taps were applied to the graduated cylinder until the powder volume changes stopped in the cylinder to obtain the tapped density. Finally, the ratio of powder mass to volume was calculated, and tapped density was obtained (Etzbach et al., 2020).

#### 2.7.5 Measurement of cohesiveness and compressibility indices

The powder cohesion or Hausner ratio (HR), according to Equation (4), and the compressibility index, according to Equation (5), were determined, respectively (Sarabandi & Sadeghi Mahoonak, 2016).

(4) $$\mathrm{HR}=\frac{\;\rho_{t}\;}{\rho_{b}\;}$$

where HR is the Hausner ratio, *ρ*_*t*_ is the density of the impact mass ($\frac{g}{{\mathrm{cm}}^{3}}$), and *ρ*_*b*_ is the density of the mass ($\frac{g}{{\mathrm{cm}}^{3}}$).

(5) $$\mathrm{CI}=\frac{\rho_{t}-\rho_{b}}{\rho_{t}}$$

CI is the compressibility index.

#### 2.7.6 Measurement of encapsulation efficiency (EE)

First, the calibration curve was plotted with different amounts of gallic acid in mg. Using an Amicon filter, the encapsulated date pit extract was separated from the free extract. One milliliter of the free extract was mixed with 1 ml of 2% sodium carbonate solution and 200 μl of Folin–Ciocalteu reagent and centrifuged for 5 min at 1200 rpm. After being at room temperature for 30 min, the absorbance of the samples was measured by a spectrophotometer at 765 nm.

Then, inserting the results in the calibration curve, the amount of total phenol in the free extract was determined in mg, gallic acid, and according to Equation (6) (Arulmozhi et al., 2013).

(6) $$\mathrm{EE}\;\left(\%\right)=\frac{C_{e}}{C_{t}}\times 100$$

where *C*_*e*_ is the amount of encapsulated phenolic compounds, and *C*_*t*_ is the amount of total phenolic compounds.

#### 2.7.7 Loading capacity (LC)

The loading rate of phenolic compounds was determined using Equation (7) (Arulmozhi et al., 2013).

(7) $$\mathrm{LC}\left(\%\right)=\frac{C_{e}}{\;C_{\text{Weight of nanoparticle}}}\times 100$$

where LC is the loading capacity (%), *C*_*e*_ is the amount of encapsulated phenolic compounds (%), and *C* is the weight of the loaded particles.

#### 2.7.8 Measurement of the release rate (Rr) for 24 h

The release rate of finely microencapsulated phenolic compounds was measured based on the Folin–Ciocalteu method. For this purpose, at certain time intervals (1, 2, 3, 4, 6, 8, 10, and 24 h), 3 g of the capsule was mixed with 3 g of phosphate buffer (pH 7), and the resulting solution was centrifuged at 4500 rpm at room temperature for 90 min Then, the supernatant was collected. The total amount of phenolic compounds was measured and stated in terms of gallic acid according to Equation (8) (Esfanjani et al., 2015).

(8) $$\mathrm{Rr}\;\left(\%\right)=\frac{\mathrm{RPC}}{\mathrm{TPC}\;}\times 100$$

where Rr is the release rate (%), RPC is the percentage of released phenolic compounds, and TPC is the percentage of total phenolic compounds.

#### 2.7.9 Morphological study of microcapsules

Scanning electron microscopy (MIRA\\LMU, TESCAN Co, The Czech Republic) was used to examine the surface morphology (Carneiro et al., 2013).

### 2.8 Statistical analysis of data

A completely randomized design with three replications was used to analyze the data obtained from the effect of different treatments on total phenolic compounds and determine the antioxidant activity of the date pit extract. Moreover, the comparison of mean data was performed by Duncan's multiple‐range test with a probability level of *α* = 5% and using SPSS software version 16. The response surface method (RSM) designs the test matrix based on the number of variables and the maximum and minimum range for each independent variable. In the design of experiments, the arrangement is such that even if the test is not repeated, reliable statistical results can be obtained. Also, this method has the ability to evaluate the interaction of parameters with each other. In the RSM, a model is defined for each dependent variable which states the main and interaction effects of the factors on each variable. In Equation 9, *Y* is the predicted response, *β*_0_ is the constant coefficient, *β*_1_, *β*_2_, *β*_3_, and *β*_4_ are linear effects, *β*_11_, *β*_22_, *β*_33_, and *β*_44_ are square effects, and *β*_12_, *β*_13_, *β*_14_, *β*_23_, *β*_24_, and *β*_34_ are mutual effects.
(9)

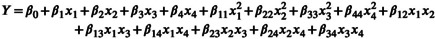

A central composite design with four independent variables, including temperature, maltodextrin concentration, *A. maurorum* gum concentration, and date pit extract concentration, was used in this study to evaluate their effect on dependent variables (response). Furthermore, the optimal operating conditions for the extraction were performed using the numerical optimization technique.

## 3 RESULTS AND DISCUSSION

### 3.1 Phenolic compounds

The results of comparing the average data obtained from different solvents on the total phenolic compounds and their antioxidant activity are shown in Table 1.

**TABLE 1:** The effect of different solvents on the total phenolic compounds and antioxidant activity of date pit extract

Treatment	Total phenolic content (mg GAE/g dm)	IC_50_ (mg/ml)
100% W	236.07 ± 9.20^d^	6.83 ± 0.18^a^
100% E	257.64 ± 14.11^c^	3.20 ± 0.13^c^
50% W:50% E	528.12 ± 25.10^b^	3.70 ± 0.11^b^
35% W:65% E	562.94 ± 22.30^b^	2.10 ± 0.08^d^
25% W:75% E	742.37 ± 18.22^a^	0.90 ± 0.01 ^e^

*Note*: Each value in the table represents the mean value of triplicate experiments. This means within each column with different letters are significantly (*p* < .05) different.

Abbreviations: E, ethanol; W: water.

According to Table 1, different solvents have a significant impact on the total amount of phenolic compounds extracted from date pit extract (*p <* .05). In this regard, the highest amount of total phenolic compounds (742.37 mg GAE/g dm) was observed in water–ethanol composite solvent (25% W: 75% E), and the lowest amount (236.07 mg GAE/g dm) in water solvent (100% W). No significant difference was observed between treatments (35% W: 65% E and 50% W: 50% E) (*p >* .05). Generally, the extraction of phenolic compounds and their antioxidant activity depends on several factors, including polarity, pH of solvents, time, extraction temperature, type, and chemical structure of phenolic compounds (Antolovich et al., 2000).

Since applying water (alone) creates a thoroughly polar environment and some phenolic compounds with a low degree of polarity are less extracted, fewer amounts of phenolic compounds extracts are extracted by water (100% W). In addition, aqueous extracts contain impurities such as organic acids, proteins, and soluble sugars that can interfere with the detection and quantification of phenolic compounds (Chirinos et al., 2007). But ethanol solution with water has a more prominent ability to extract phenolic compounds since they extract both polar and nonpolar compounds. However, the higher amount of ethanol than water in the solution (25% W: 75% E) has a more significant effect on the extraction of phenolic compounds since increasing the amount of ethanol reduces the dielectric constant of the solution, which in turn reduces the energy required for separation of solvent molecules. Thus, solute molecules are more simply placed between solvent molecules and dissolved (Pompeu et al., 2009). Similar results have been reported in the extraction of phenolic compounds from blackberry (Cacace & Mazza, 2003) and pomegranate peel (Wissam et al., 2012).

### 3.2 DPPH free radical scavenging

According to Table 1, different solvents have a significant effect on the free radical scavenging power of date pit extracts (*p <* .05). In this regard, the highest value of IC_50_ (6.83 μg/ml) was observed in water solvent (100% W), while the lowest value (0.90 μg/ml) was observed in water–ethanol combined solvent (25% W: 75% E) (*p <* .05). In other words, a significant decrease in IC_50_ was observed with increasing the amount of ethanol solvent. At a ratio of 75% ethanol, the IC_50_ value reached its lowest value, and the extraction of antioxidant compounds increased. The scavenging power of different extracts depends on the number and position of hydroxyl groups and the molecular weight of phenolic compounds (Pompeu et al., 2009). In lower‐molecular‐weight phenolic compounds, hydroxyl groups are more readily available. In addition, after hydrogen donation, phenolic compounds are converted into phenoxyl free radicals. The stability degree of these radicals can affect the antioxidant capacity of phenolic compounds, as less stable phenoxy radicals compete with DPPH radicals for the adsorption of hydrogen atoms, and therefore the trapping percentage of DPPH radicals is reduced (Chirinos et al., 2007). According to the results, each compound with more phenolic compounds showed more scavenging power. Other studies have shown a direct relationship between phenolic content and antioxidant capacity (Jimoh et al., 2010). According to some other studies, since a number of phenolic compounds have antiradical power and, in some cases, other substances also have antiradical properties, the relationship between phenolic compounds and free radical scavenging power is not necessarily direct (Chandini et al., 2008).

### 3.3 Evaluation of physicochemical properties of microcapsules

The levels of independent variables in the real and coded form are presented in Table 2. On the other hand, the analysis of the estimated regression coefficients in the second‐order polynomial model for the response variables is presented in Table 3. The positive sign of the estimated regression coefficients obtained from the central composite design model means the direct effect of the independent variables on the response variables.

**TABLE 2:** Independent variables and their values

	Code and levels				
Independent variables	−2	−1	0	+1	+2
Temperature (°C)	38	40	42	44	46
Maltodextrin concentration (%)	10	15	20	25	30
Taranjebin concentration (%)	10	15	20	25	30
Date pit extract concentration (%)	30	35	40	45	50

**TABLE 3:** Regression coefficients for prediction of the moisture, solubility, and bulk density

Regression coefficients	Moisture (%)	*F*‐value	*p*‐value	Solubility (%)	*F*‐value	*p*‐value	Bulk density (g/cm‐3)	*F*‐value	*p*‐value	Tapped density (g/cm‐3)	*F*‐value	*p*‐value
*β*_0_	5.16	90.77**	<.0001	61.26	23.35**	<.0001	0.0314	29.75**	<.0001	0.0098	65.54**	<.0001
*β*_1_	49.12	863.25**	<.0001	340.45	129.77**	<.0001	23.0910	201.36**	<.0001	0.0539	74.15**	<.0001
*β*_2_	0.0561	0.9857^ns^	.3442	5.34	2.03^ns^	.1757	0.0325	0.0101^ns^	.9214	0.0051	22.52**	<.0001
*β*_3_	2.53	44.49**	<.0001	60.39	23.02**	.0003	36.0243	53.84**	<.0001	0.0070	2.49^ns^	.1542
*β*_4_	0.0065	0.1147^ns^	.7419	63.90	24.36**	.0002	0.0541	0.0086^ns^	.9275	0.0137	0.5214^ns^	.2430
*β*_12_	0.0612	1.08^ns^	.3242	3.27	2.77^ns^	.1181	0.0000	0.0446^ns^	.8359	0.0433	32.12**	<.0001
*β*_13_	0.4704	8.27*	.0165	31.04	5.0174 **	.8969	18.0030	6.69**	<.0001	0.0057	2.123^ns^	.021
*β*_14_	0.0495	0.8694^ns^	.3731	27.79	3.3017 **	.5915	0.0012	2.57^ns^	.1311	5.300E−06	0.541^ns^	.1251
*β*_23_	0.1310	2.30^ns^	.1601	0.1090	0.0416^ns^	.8414	0.0005	1.12^ns^	.3087	0.0219	1.42^ns^	.245
*β*_24_	0.1029	1.81^ns^	.2085	0.1769	0.0674^ns^	.7989	0.0013	2.95^ns^	.1080	0.0003	1.623^ns^	.0421
*β*_34_	0.0320	0.5631^ns^	.4703	2.224	29.06^ns^	<.0001	0.0009	1.88^ns^	.1914	0.0123	26.34**	<.0001
*β*_11_	2.56	45.05**	<.0001	2.56	5.05^ns^	<.0001	2.126	1.94^ns^	.2514	1.360	2.75^ns^	.875
*β*_22_	0.0546	0.9598^ns^	.3503	0.3691	1.402^ns^	.0872	0.2587	0.418^ns^	.0751	0.0985	1.412^ns^	.0214
*β*_33_	0.2947	5.18*	.0461	0.1760	0.125^ns^	.0341	0.1548	2.139^ns^	.1254	0.0452	2.502^ns^	.1492
*β*_44_	0.0004	0.0072^ns^	.9340	0.0213	0.0428^ns^	.0685	0.0845	0.0485^ns^	.0432	0.0231	0.042^ns^	.9750
*R* ^2^	0.988	–	–	0.964	–	–	0.981	–	–	0.998	–	–
Adj‐*R* ^2^	0.8735	–	–	0.883	–	–	0.896	–	–	0.8874	–	–

Regression coefficients	Compressibility (%)	*F*‐value	*p*‐value	Total phenolic (g/mg Gallic acid)	*F*‐value	*p*‐value	Loading capacity	*F*‐value	*p*‐value
*β*_0_	0.1425	6.36**	<.0001	88498.71	4.09**	<.0001	479.26	3.80**	.0198
*β*_1_	0.2137	9.54**	<.0001	1.772E+05	8.19**	<.0001	995.68	7.90**	.0184
*β*_2_	0.1093	4.88**	<.0001	20,304.02	0.9381**	<.0001	102.48	0.8133^ns^	.3884
*β*_3_	0.1335	5.96**	<.0001	2943.53	0.1360**	<.0001	162.77	1.29^ns^	.2822
*β*_4_	0.5757	25.70**	<.0001	1.263E+05	5.83**	<.0001	503.24	3.99**	.0736
*β*_12_	0.0035	4.40**	<.0001	178.69	0.0083	.9294	1.54	0.0122^ns^	.9141
*β*_13_	0.0987	1.302^ns^	<.0001	1.210E+05	5.59	.0396	756.04	6.00**	.0343
*β*_14_	0.4230	2.012	.5922	1.359E+05	6.28**	<.0001	729.69	5.79**	.0369
*β*_23_	0.0292	1.30	.8653	1564.41	0.0723**	<.0001	8.10	0.0643^ns^	.8050
*β*_24_	0.0372	1.16	.7231	481.83	0.0223	.8844	10.52	0.0835^ns^	.7786
*β*_34_	0.2070	9.24**	<.0001	1.595E+05	7.37	.0218	855.20	6.79^ns^	.7263
*β*_11_	0.847	1.05^ns^	.0557	60,493.43	2.79	.1255	384.94	3.05**	.1111
*β*_22_	0.214	0.402^ns^	.0854	17,108.21	0.7905	.3948	99.35	0.7884^ns^	.3954
*β*_33_	0.0563	0.544^ns^	.0257	1667.33	0.0770	.7870	24.36	0.1933^ns^	.6695
*β*_44_	0.0843	0.021^ns^	.0563	2.433E+05	11.24	.0073	1284.53	10.19**	.0096
*R* ^2^	0.752	–	–	0.983	–	–	0.979	–	–
Adj‐*R* ^2^	0.236	–	–	0.888	–	–	0.895	–	–

*Note*: *β*_1_ = Temperature, *β*_2_ = Date kernel concentration, *β*_3_ = Maltodextrin concentration, and *β*_4_ = Taranjebin concentration, *Significant at .05 level, **Significant at .01 level, and ns: Not significant.

#### 3.3.1 Evaluating the moisture content

**FIGURE 2:**
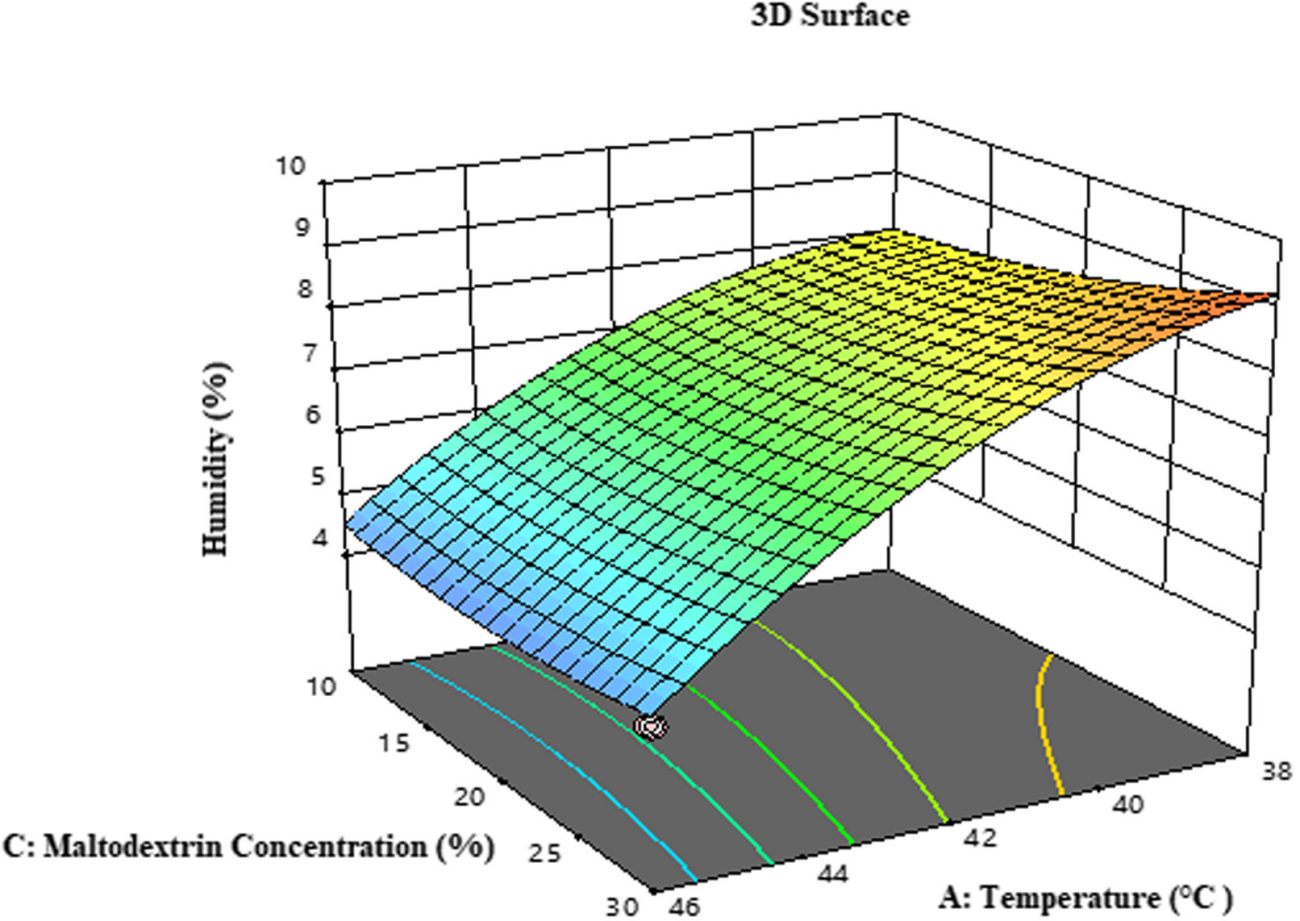
Mutual effect of temperature and maltodextrin concentration on the moisture content of microcapsules.

According to Table 3, the effect of temperature (*β*_1_), maltodextrin concentration (*β*_3_), the second‐order mutual effect of temperature (*β*_11_) (*p <* .01), the mutual effect of temperature and maltodextrin concentration (*β*_13_), and the second‐order mutual effect of maltodextrin concentration (*β*_33_) (*p <* .05) was significant on the moisture content of microcapsules. The high coefficient of determination (*R* ^2^) and the amounts of *F*‐value and *p*‐value indicate that the proposed model has an optimal fit to determine the moisture content of the samples. The physicochemical stability of powders during storage depends on the moisture content of the powder. To increase the storage time, the moisture content of the samples should be <4%–5% (Sarabandi & Sadeghi Mahoonak, 2016). The low moisture content of the powder prevents the degradation of the microcapsule active compounds in the powder structure. As Figure 2 shows, the moisture content of the powders decreased by increasing the temperature of the dryer inlet air. The result is that increasing the temperature difference between atomized particles and the drying medium increases the simultaneous transfer rate of mass and energy, which results in more moisture released from the powder particles (Oberoi & Sogi, 2015; Santhalakshmy et al., 2015). Increasing the concentration of maltodextrin, the moisture content of the powder samples increased. The cause of this phenomenon can be related to the tendency of maltodextrin sugars to absorb moisture and increase viscosity, thus reducing the free moisture in powders for evaporation. Large maltodextrin molecules are also a barrier to the easy diffusion of water molecules (Sarabandi & Sadeghi Mahoonak, 2016).

#### 3.3.2 Measurement of the solubility percentage

**FIGURE 3:**
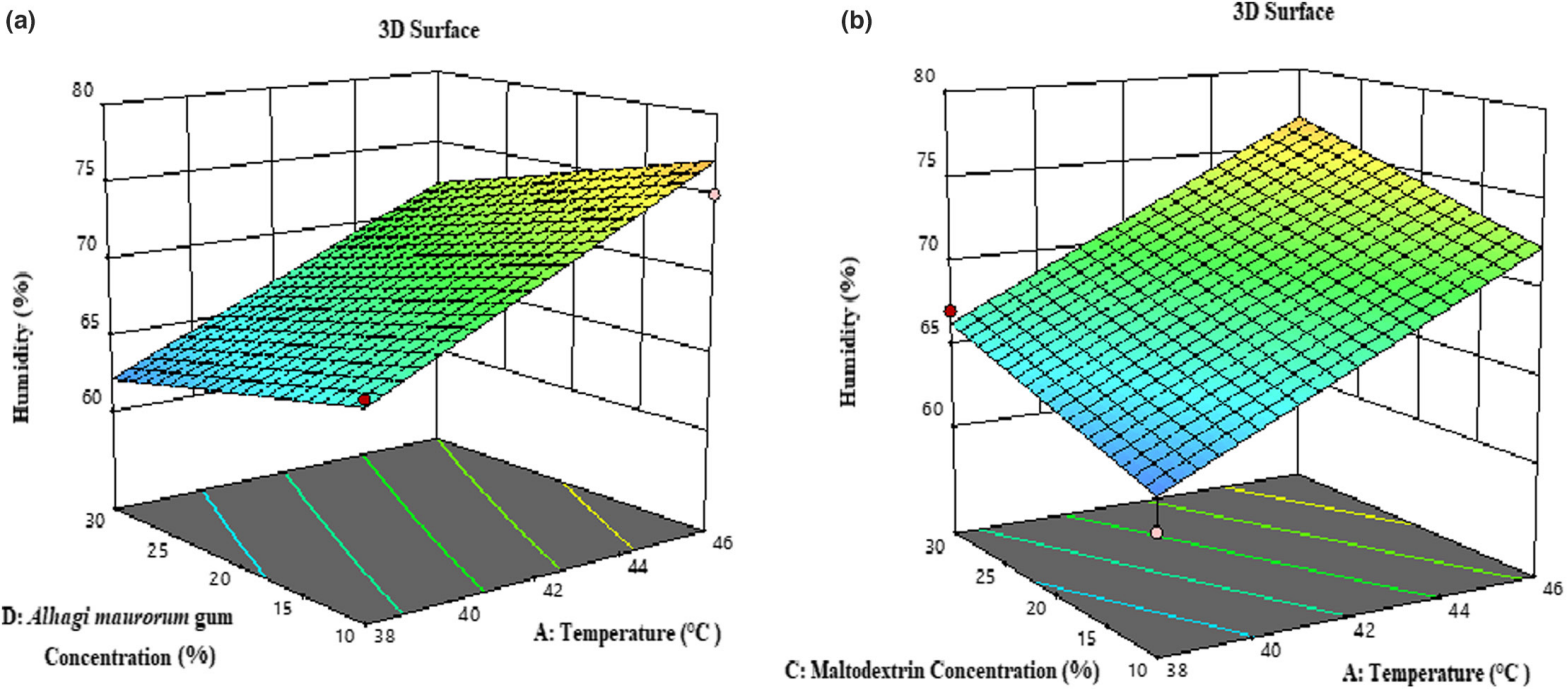
3‐D surface plot showing the effects of temperature and *Alhagi maurorum* gum concentration (a); temperature and concentration of maltodextrin (b) on the solubility of microcapsules.

Based on Table 3, the effect of temperature (*β*_1_), maltodextrin concentration (*β*_3_), *A. maurorum* concentration (*β*_4_), and the mutual effect of temperature with maltodextrin (*β*_13_) as well as temperature with *A. maurorum* gum (*β*_14_) on the solubility of microcapsules is significant (*p* < .01). The high coefficient of determination (*R* ^2^) and the amount of *F*‐value and *p*‐value indicate that the proposed model has an optimal fit to determine the solubility of the samples. The solubility of the powder is an important property that affects its behavior when dissolved in water. Factors such as size, shape, composition, surface properties, type and composition of raw material, type of feed (solid concentration), and drying conditions affect the solubility of powders. Powder rehydration in the water process is divided into four stages: wetting, immersion, dispersion, and dissolution (Kim et al., 2005). According to Figure 3a,b, with rising inlet air temperature in the dryer chamber, the solubility of powders increased due to the decrease in moisture content (Santhalakshmy et al., 2015). Walton (2000) showed that the particle size increased as the inlet air temperature increased. Consequently, the required time for re‐dewatering was reduced. Increasing the inlet air temperature increases the apparent porosity and thus improves the susceptibility of the particles. However, a study by Quek et al. (2007) showed that increasing the dryer temperature reduces the solubility of the powder. The researchers attributed the result to the formation of a hard layer on the surface of the particles, preventing water molecules from penetrating the particles and thus reducing the wetting capacity of the particles. According to Figure 3a,b, increased the solubility of powders were increased by increasing the concentration of maltodextrin and *A. maurorum* gum. Maltodextrin has appropriate physical properties and high solubility due to the presence of hydrophilic groups in its molecular structure (Goula & Adamopoulos, 2010; Zendeboodi et al., 2018).

It is also possible to increase the solubility of powders by increasing the size and space between particles and subsequently facilitating the penetration of moisture into the structure of powders, which occurs due to increasing the concentration of maltodextrin (Sarabandi & Sadeghi Mahoonak, 2016). However, some studies have reported a decrease in the solubility percentage with increasing concentrations of maltodextrin (Cano‐Chauca et al., 2005; Mahendran, 2010).

#### 3.3.3 Bulk density measurement

Based on Table 3, temperature (*β*1), maltodextrin concentration (*β*3), and the mutual effect of temperature with maltodextrin (*β*13) have a significant impact on the bulk density of microcapsules (*p <* .01). The high coefficient of determination (*R* ^2^) and the amount of *F*‐value and *p*‐value indicate the optimal fit of the proposed model. Bulk density depends on the size and shape of the particle, moisture, chemical composition, and the amount of air trapped inside the particle.

**FIGURE 4:**
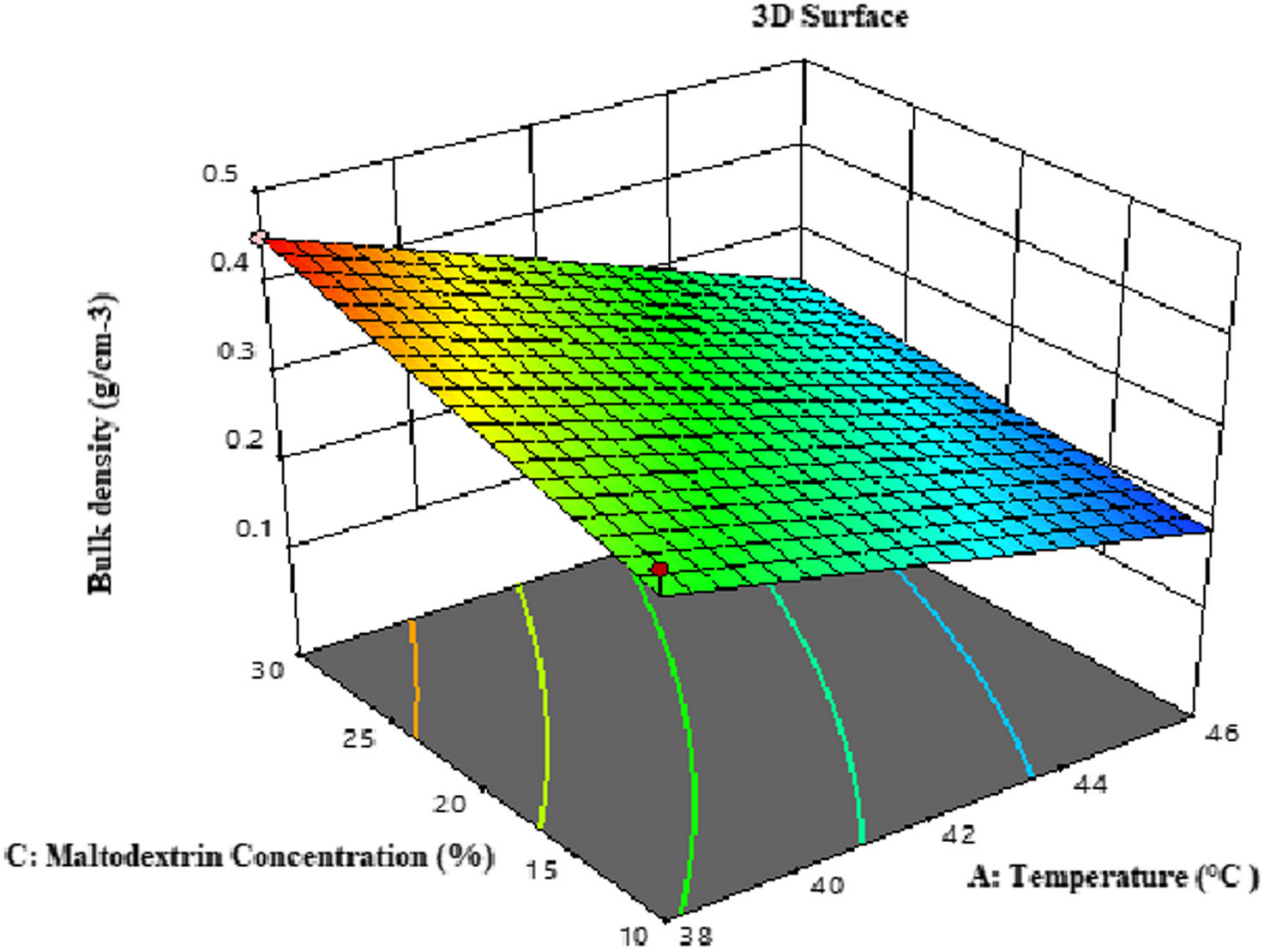
3‐D surface plot showing the effects of temperature and maltodextrin concentration on the bulk density of microcapsules.

These factors depend on feed characteristics, inlet air volume, temperature and drying time, processing, and transportation operations (Goula & Adamopoulos, 2010). According to Figure 4, the density of the mass decreased with increasing inlet air temperature. Because of increasing temperature, the rate of moisture evaporation increases. As a result, the size of the particles becomes larger, and their weight decreases. Also, more spherical particles are produced (Goula & Adamopoulos, 2010). Increasing maltodextrin concentration, the bulk density increased due to the higher bulk mass due to the moisture (Sarabandi & Sadeghi Mahoonak, 2016). The particles tend to stick together with increasing humidity. Thus, the space between the particles decreases, and a more significant amount of powder occupies a certain volume of space, which can also be a reason for increasing bulk density (Goula & Adamopoulos, 2010; Zendeboodi et al., 2018).

#### 3.3.4 Tapped density

**FIGURE 5:**
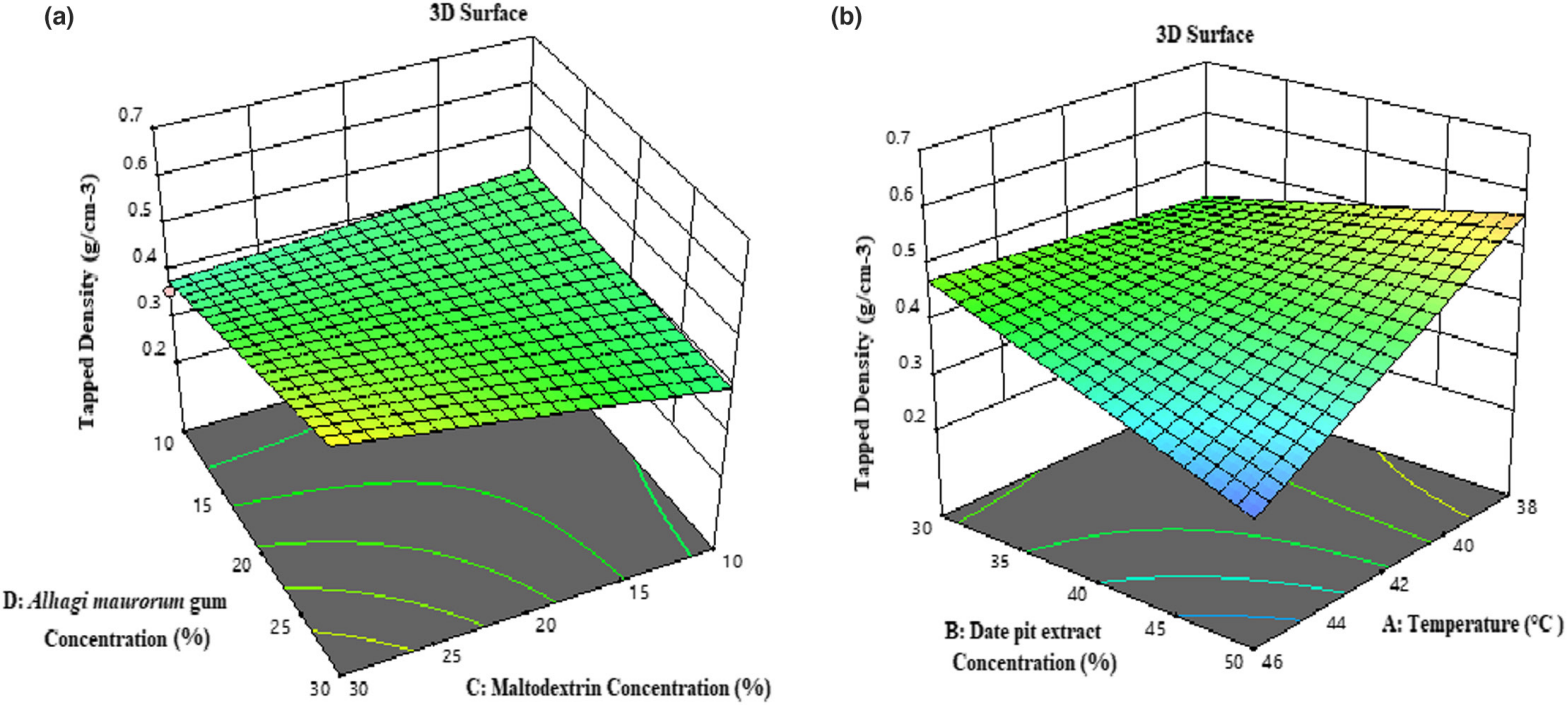
3‐D surface plot showing the effects of maltodextrin concentration and *Alhagi maurorum* concentration (a); temperature and date pit extract concentration (b) on the tapped density of microcapsules.

According to Table 3, the effect of temperature (*β*_1_), the concentration of date pit extract (*β*_2_), the mutual effect of temperature with date pit extract concentration (*β*_12_), and the mutual effect of maltodextrin concentration with *A. maurorum* gum concentration (*β*_34_) are significant on the tapped density of capsules (*p <* .01). The high coefficient of determination (*R* ^2^) and the amount of *F*‐value and *p*‐value indicate the optimal fit of the proposed model. Tapped density is a critical indicator in the packaging and transportation process which determines the amount of material needed to fill a certain volume of packages, and storage containers as well. According to Figure 5a,b, the tapped density of the samples decreased with increasing temperature, the concentration of date pit extract, the concentration of maltodextrin, and *A. maurorum* gum. The results indicated that with increasing temperature, the moisture content of powder samples decreases, and as a result, the particles become lighter, and the density decreases (Goula & Adamopoulos, 2010; Zendeboodi et al., 2018). The decrement of tapped density with increasing maltodextrin concentration is due to the particular properties of maltodextrin, which reduces the adhesion between particles (Peighambardoust & Sarabandi, 2017). Increasing the concentration of maltodextrin and *A. maurorum* gum increases water absorption because the powder particles become larger and more spherical, and the space between the particles is slightly filled with air. This process is an effective factor in reducing tapped density. As mentioned, particle size is a factor influencing tapped density. If the percentage of coarse particles in the powder increases, the volume does not change much due to the tapping. Thus, the tapped density decreases. Increasing the concentration of carriers and date pit extract leads to producing larger droplets and eventually larger dried particles in the drying chamber (Goula & Adamopoulos, 2010; Kha et al., 2010).

#### 3.3.5 Compressibility index

**FIGURE 6:**
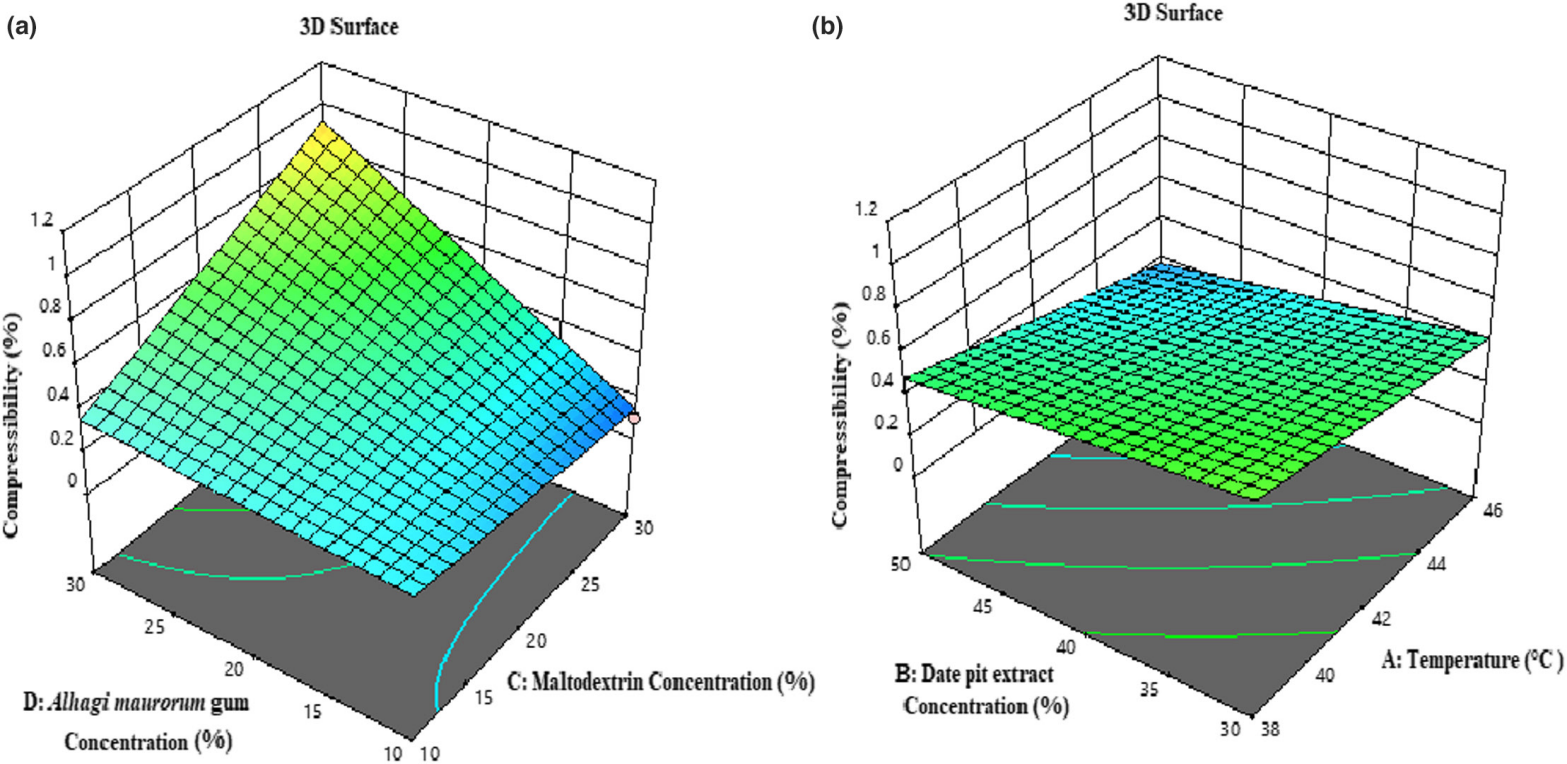
3‐D surface plot showing the effects of maltodextrin concentration and *Alhagi maurorum* concentration (a); temperature and date pit extract concentration (b) on the compressibility index of microcapsules.

According to Table 3, the effect of temperature (*β*_1_), date pit extract concentration (*β*_2_), maltodextrin concentration (*β*_3_), *A. maurorum* gum concentration (*β*_4_), temperature mutual effect with date pit extract concentration (*β*_12_), and mutual effect of maltodextrin concentration with *A. maurorum* gum concentration (*β*_34_) is significant on the compressibility index of microcapsules (*p <* .01). Alternately, the values of coefficient of determination (*R* ^2^), *F*‐value, and *p*‐value indicate that the proposed model has an optimal fit. Compressibility is one of the most essential and influential properties of powder handling and final processing. It is a function of the powder's physical properties, such as particle size, shape, surface structure, particle density, bulk density, moisture content, temperature, pressure, and fat (Kim et al., 2005). According to Figure 6a,b, with increasing temperature and concentration of date pit extract, the compressibility index of the samples decreased because of increasing temperature, and the formation of liquid relations between the powder particles decreased. Furthermore, increasing the concentration of the extract, the particle size and the empty spaces between them increase (Peighambardoust & Sarabandi, 2017). On the other hand, this index increased with increasing concentrations of maltodextrin and *A. maurorum* gum. The result is that increasing the moisture softens and plasticizes the powder components, especially water‐soluble components, which causes deformation and provides a higher contact surface (Kim et al., 2005).

#### 3.3.6 Evaluating encapsulation efficiency

**FIGURE 7:**
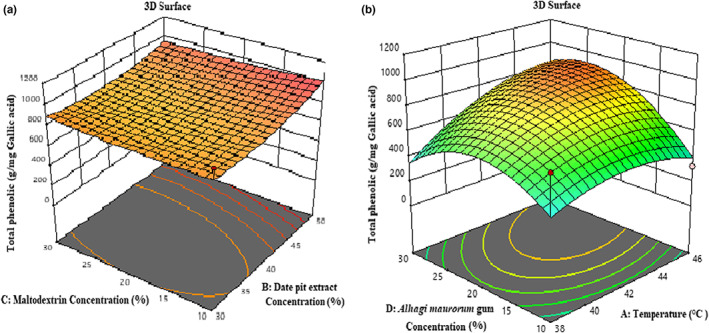
3‐D surface plot showing the effects of maltodextrin concentration and date pit extract concentration (a); temperature and *Alhagi maurorum* concentration (b) on the encapsulation efficiency of microcapsules.

According to Table 3, the effect of temperature (*β*_1_), date pit extract concentration (*β*_2_), maltodextrin concentration (*β*_3_), *A. maurorum* gum concentration (*β*_4_), temperature mutual effect with *A. maurorum* gum concentration (*β*_14_), and mutual effect of maltodextrin concentration with date pit extract concentration (*β*_32_) is significant on the encapsulation efficiency of microcapsules (*p <* .01). According to Figure 7b, the total phenolic compounds increased with increasing inlet air temperature to 42°C and then decreased; by increasing the temperature to 42°C, capsule formation was done well and more extracts were coated. But a further increase in temperature could destroy the structure of the phenolic compound due to enzymatic decomposition or thermal degradation (Akbarbaglu et al., 2018). The study of Goula and Adamopoulos (2010) showed that by increasing the air temperature entering the dryer, the amount of lycopene in tomato powder decreases. Similar results have been observed in the research of other researchers (Goula & Adamopoulos, 2010). Additionally, according to Figure 7a,b, the total amount of phenolic compounds increased and then decreased with increasing concentrations of maltodextrin and *A. maurorum* gum (up to 20%). The reason for the increase in phenolic compounds is the protective effect of these coatings at high temperatures, and conversely, the reason for the decrease in phenolic compounds is the increase in the mass of nonphenolic carriers (coatings) (Moser et al., 2017; Tolun et al., 2016).

#### 3.3.7 Evaluating loading capacity

**FIGURE 8:**
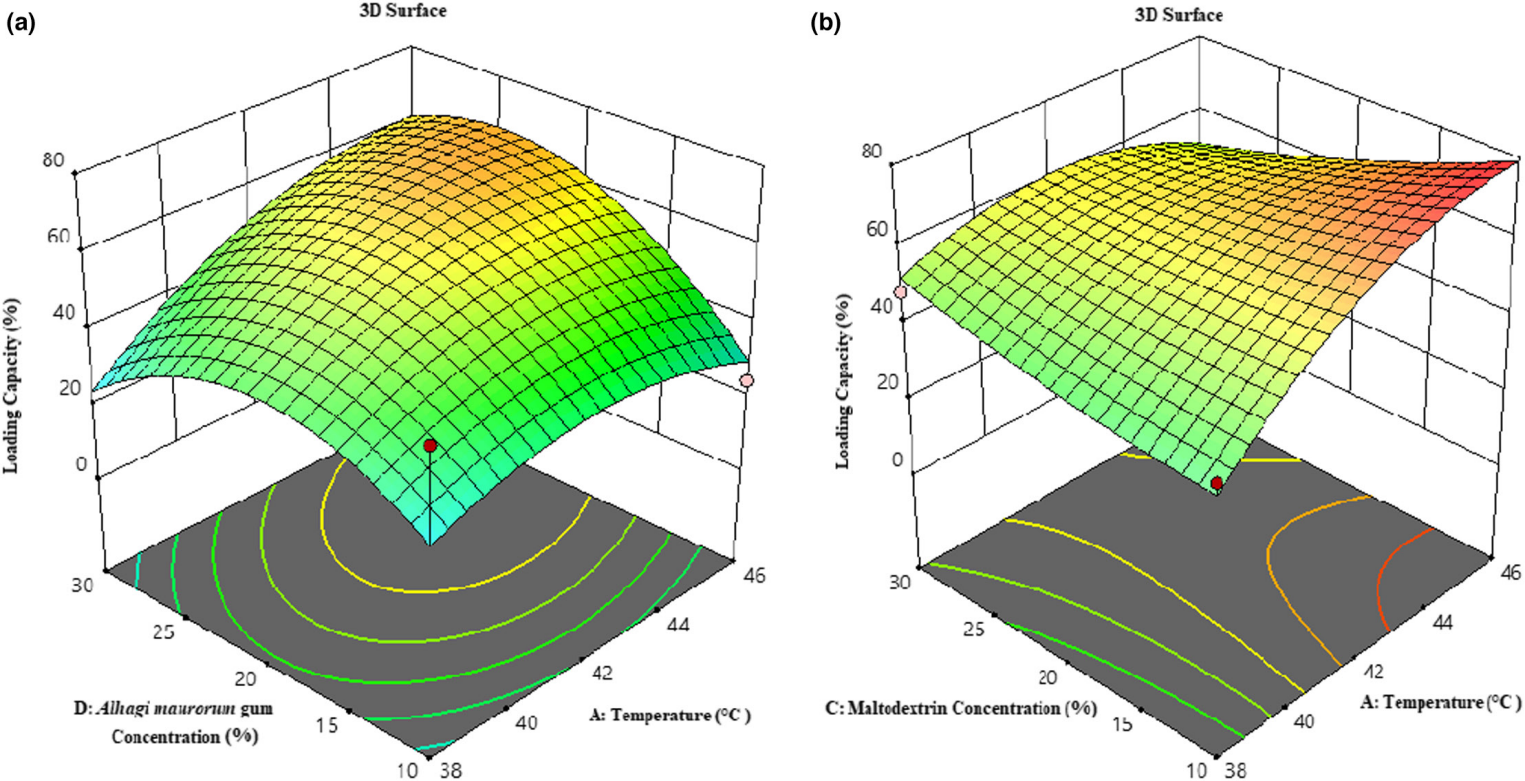
3‐D surface plot showing the effects of temperature and Alhagi maurorum gum concentration (a); temperature and concentration of maltodextrin (b) on the loading capacity of microcapsules.

According to Table 3, temperature (*β*_1_), the concentration of *A. maurorum* gum (*β*_4_), the mutual effect of temperature with the concentration of maltodextrin (*β*_13_), the mutual effect of temperature with the concentration of *A. maurorum* gum (*β*_14_), the mutual effect of the second order of temperature (*β*_11_), and the mutual effect of the second order of the *A. maurorum* gum concentration (*β*_44_) significantly affected the loading capacity of microcapsules (*p <* .01). Loading capacity is the capacity of carriers to store and then release active compounds. This feature is affected by the capsule preparation process, sample volume, temperature, and surface properties of the sample. According to Figure 8a,b, the loading capacity increased with increasing inlet air temperature to 42°C and then decreased; by increasing the temperature to 42°C, capsule formation was well done, and more extracts were coated. But a further increase in temperature could destroy the structure of the phenolic compound due to enzymatic decomposition or thermal degradation (Akbarbaglu et al., 2018). Besides, the loading capacity increased with increasing concentrations of maltodextrin and *A. maurorum* gum (up to 20%) and then decreased. The reason for increasing phenolic compounds is the protective effect of these coatings at high temperatures, in contrast, the increase in nonphenolic carriers (coatings) mass is the reason for the phenolic compounds decrement (Moser et al., 2017; Tolun et al., 2016).

#### 3.3.8 Release rate

**FIGURE 9:**
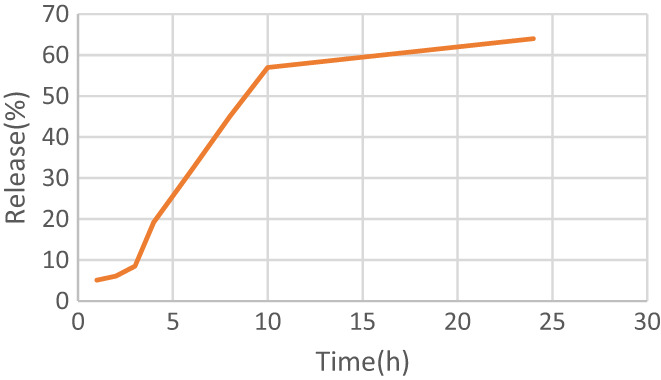
Release of phenolic compounds from loaded microcapsules.

The release of phenolic compounds from date pit extract was investigated at intervals of 1, 2, 3, 4, 6, 8, 10, and 24 h. According to Figure 9, the release of phenolic compounds reached its maximum value (64%) 24 h after the experiment. Increasing the release of effective materials reduces the stability and cohesion of biopolymers used as a coating material (wall). It increases the release rate under environmental stresses such as temperature and humidity. The absorption of moisture from the environment causes the biopolymer wall to swell, and at the same time, the glass transfer temperature decreases. As a result, the cohesiveness and entanglement of the polymer chains are reduced, and the finely divided particles' rate of molecular motion increases. Under these conditions, due to the breakdown of raw material tissue, the effective diffusion coefficient of these compounds from inside the microcapsules increases, and their reduction intensifies over time (Najafi et al., 2011). Similar results have been reported in various studies (Akbarbaglu et al., 2018; Arulmozhi et al., 2013; Goula & Adamopoulos, 2010; Kha et al., 2010).

#### 3.3.9 Morphological evaluation of microcapsules

**FIGURE 10:**
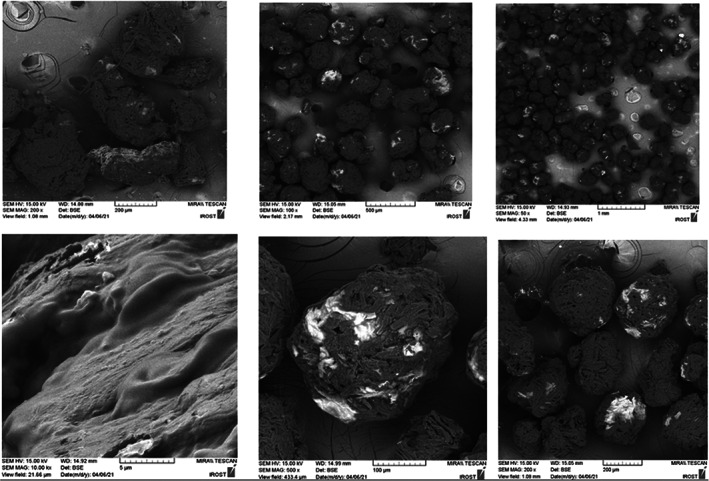
SEM micrographs of date pit phenolic compounds microencapsulated powders produced by maltodextrin (20 % W/V) as the first wall, *Alhagi maurorum* gum (20 % W/V) for the second wall, and MCT oil (15% W/W) as the third wall.

Figure 10 shows the optimal effect of temperature (45°C), maltodextrin (20%), and *A. maurorum* gum (20%) on the surface structure and morphological characteristics of the powder‐containing date pit extract dried applying the fluidized‐bed method. The presence of depressions and cavities in the surface of microcapsules is related to the low viscoelastic properties of *A. maurorum* gum, which is not able to withstand the stresses caused by the rapid outflow of water in the early stages of drying and has caused cavities. Surface wrinkles and cavities on the microcapsule surface may also occur because of the mechanical stresses due to drying conditions on the wall material.

## 4 CONCLUSIONS

In this study, the effect of different solvents on the total phenolic compound extracted from date pit extract and the physicochemical properties of microcapsules produced at different temperatures and concentrations of maltodextrin and *A. maurorum* gum as a coating by fluidized‐bed dryer were investigated. According to the results, the type of solvents and their polarity in the extraction of phenolic compounds significantly affected the extraction efficiency. In this regard, the highest and lowest amount of total phenolic compound was extracted in a water–ethanol mixed solvent (25% W: 75% E) and water (100% W). The ethanol and water mixture had a more impressive ability to extract phenolic compounds than any solvents alone and this effect was more significant in higher percentages of ethanol. On the other hand, a direct relationship between free radical inhibition and the amount of total phenolic compound was observed. The highest value of IC_50_ was observed in a water solvent (100% W), and the lowest value was observed in a water–ethanol mixed solvent (25% W: 75% E).

Notably, in solvents containing 75% ethanol, the IC_50_ reached its lowest value, and the extraction of antioxidant compounds increased. Also, all the physicochemical properties of microcapsules‐related tests, including moisture, bulk density, loading capacity, impact density, compressibility, and functionality, such as solubility and microencapsulation efficiency, were significantly dependent on process conditions, such as carrier concentrations and process temperature. Excessive increase in carrier ratio and process temperature due to adverse effects on the drying process and increase in viscosity caused the quantitative and qualitative loss of microcapsules. The optimal physicochemical properties of the microcapsules were obtained at 45°C and 20% of each carrier (maltodextrin and tangerine). The release of phenolic compounds increased after 24 h due to the decrease in stability and cohesion of the biopolymer compounds used as coating material and the effect of environmental stresses, such as temperature and heat. The surface structure of particles and the SEM results also showed the surface properties and uniformity of the particles under the influence of the concentration of maltodextrin and *A. maurorum* gum and the process temperature. Overall, the research results show that the use of maltodextrin and *A. maurorum* gum carriers and a fluid bed dryer can be effective as a promising method in increasing the stability of microencapsulated date pit extract against environmental conditions. Also, microencapsulated compounds can be used in a wide range of bakery products, oil, etc., to improve their quality characteristics and extend their shelf life.

## FUNDING INFORMATION

This research received no specific grant from any funding agency in the public, commercial, or not‐for‐profit sectors.

## CONFLICT OF INTEREST

The authors declare that they do not have any conflict of interest.

## Data Availability

Research data are not shared.

## References

[fsn33173-bib-0001] Akbarbaglu, Z. , Peighanbardoust, S. , Oladgaffari, A. , & Sarabandi, K. (2018). Effect of inlet air temperature and carrier type and concentration on physicochemical and antioxidant properties of microencapsulated marjoram extract by spray drying. Journal of Food Research, 28(4), 15–30. [In Persian].

[fsn33173-bib-0002] Antolovich, M. , Prenzler, P. , Robards, K. , & Ryan, D. (2000). Sample preparation in the determination of phenolic compounds in fruits. The Analyst, 125(5), 989–1009.

[fsn33173-bib-0003] Arulmozhi, V. , Pandian, K. , & Mirunalini, S. (2013). Ellagic acid encapsulated chitosan nanoparticles for drug delivery system in human oral cancer cell line (KB). Colloids and Surfaces B: Biointerfaces, 110, 313–320. 10.1016/j.colsurfb.2013.03.039 23732810

[fsn33173-bib-0004] Awulachew, M. T. (2021). A systematic review of encapsulation and control release technology in food application. International Journal of Food Science and Technology, 7(3), 292–296. 10.17352/2455-815x.000122

[fsn33173-bib-0005] Baliga, M. S. , Baliga, B. R. V. , Kandathil, S. M. , Bhat, H. P. , & Vayalil, P. K. (2011). A review of the chemistry and pharmacology of the date fruits (*Phoenix dactylifera* L.). Food Research International, 44, 1812–1822. 10.1016/j.foodres.2010.07.004

[fsn33173-bib-0006] Başyiğit, B. , Sağlam, H. , Kandemir, Ş. , Karaaslan, A. , & Karaaslan, M. (2020). Microencapsulation of sour cherry oil by spray drying: Evaluation of physical morphology, thermal properties, storage stability, and antimicrobial activity. Powder Technology, 364, 654–663. 10.1016/j.powtec.2020.02.035

[fsn33173-bib-0007] Budinčić, J. M. , Petrović, L. , Đekić, L. , Fraj, J. , Bučko, S. , Katona, J. , & Spasojević, L. (2021). Study of vitamin E microencapsulation and controlled release from chitosan/sodium lauryl ether sulfate microcapsules. Carbohydrate Polymers, 251, 116988. 10.1016/j.carbpol.2020.116988 33142560

[fsn33173-bib-0008] Cacace, J. E. , & Mazza, G. (2003). Mass transfer process during extraction of phenolic compounds from milled berries. Journal of Food Engineering, 59(4), 379–389. 10.1016/S0260-8774(02)00497-1

[fsn33173-bib-0009] Cano‐Chauca, M. , Stringheta, P. C. , Ramos, A. M. , & Cal‐Vidal, J. (2005). Effect of the carriers on the microstructure of mango powder obtained by spray drying and its functional characterization. Innovative Food Science and Emerging Technologies, 6(4), 420–428. 10.1016/j.ifset.2005.05.003

[fsn33173-bib-0010] Carneiro, H. C. , Tonon, R. V. , Grosso, C. R. , & Hubinger, M. D. (2013). Encapsulation efficiency and oxidative stability of flaxseed oil microencapsulated by spray drying using different combinations of wall materials. Journal of Food Engineering, 115(4), 443–451. 10.1016/j.jfoodeng.2012.03.033

[fsn33173-bib-0011] Chandini, S. K. , Ganesan, P. , & Bhaskar, N. (2008). In vitro antioxidant activities of three selected brown seaweeds of India. Food Chemistry, 107(2), 707–713. 10.1016/j.foodchem.2007.08.081

[fsn33173-bib-0012] Chao, C. T. , & Krueger, R. R. (2007). The date palm (*Phoenix dactylifera* L.): Overview of biology, uses, and cultivation. HortScience, 42(5), 1077–1082. 10.21273/HORTSCI.42.5.1077

[fsn33173-bib-0013] Chirinos, R. , Rogez, H. , Campos, D. , Pedreschi, R. , & Larondelle, Y. (2007). Optimization of extraction conditions of antioxidant phenolic compounds from mashua (*Tropaeolum tuberosum* Ruíz & Pavón) tubers. Separation and Purification Technology, 55(2), 217–225. 10.1016/j.seppur.2006.12.005

[fsn33173-bib-0014] Dobrinčić, A. , Tuđen, L. , Repajić, M. , Garofulić, I. E. , Zorić, Z. , Dragović‐Uzelac, V. , & Levaj, B. (2020). Microencapsulation of olive leaf extract by spray drying. Acta Alimentaria, 49(4), 475–482. 10.1556/066.2020.49.4.13

[fsn33173-bib-0015] El‐Massry, K. , El‐Ghorab, A. , Farouk, A. , Hamed, S. , Elgebaly, H. , Mosa, N. , & Mahmoud, A. (2019). Microencapsulation of date seed oil by spray‐drying for stabilization of olive oil as a functional food. Asian Journal of Scientific Research, 12, 516–523. 10.3923/ajsr.2019.516.523

[fsn33173-bib-0016] Esfanjani, A. F. , Jafari, S. M. , Assadpoor, E. , & Mohammadi, A. (2015). Nano‐encapsulation of saffron extract through double‐layered multiple emulsions of pectin and whey protein concentrate. Journal of Food Engineering, 165, 149–155. 10.1016/j.jfoodeng.2015.06.022

[fsn33173-bib-0017] Etzbach, L. , Meinert, M. , Faber, T. , Klein, C. , Schieber, A. , & Weber, F. (2020). Effects of carrier agents on powder properties, stability of carotenoids, and encapsulation efficiency of goldenberry (*Physalis peruviana* L.) powder produced by co‐current spray drying. Current Research in Nutrition and Food Science Journal, 3, 73–81. 10.1016/j.crfs.2020.03.002 PMC7473355 32914123

[fsn33173-bib-0018] FAOSTAT . (2019). FAO. FAOSTAT statistics division of the food and agriculture organization of the United Nations. http://www.fao.org/faostat/en/#data

[fsn33173-bib-0019] Goula, A. M. , & Adamopoulos, K. G. (2010). A new technique for spray drying orange juice concentrate. Innovative Food Science and Emerging Technologies, 11(2), 342–351. 10.1016/j.ifset.2009.12.001

[fsn33173-bib-0020] Habib, M. H. , & Ibrahim, H. W. (2009). Nutritional quality evaluation of eighteen date pit varieties. International Journal of Food Sciences and Nutrition, 60(1), 99–111. 10.1080/09637480802314639 18925479

[fsn33173-bib-0021] Hameed, A. , Hussain, S. A. , Nosheen, S. , Muhammad, Z. , Wu, Y. , Ullah, S. , & Song, Y. (2020). Microencapsulation of microbial antioxidants from *Mucor circinelloides*, their physico‐chemical characterization, in vitro digestion and releasing behaviors in food. Applied Biological Chemistry, 63, 1–17. 10.1186/s13765-020-00512-2

[fsn33173-bib-0022] Hasheminya, S. M. , & Dehghannya, J. (2020). Composition, phenolic content, antioxidant and antimicrobial activity of *Pistacia atlantica* subsp. *kurdica hulls'* essential oil. Food Bioscience, 34, 100510. 10.1016/j.fbio.2019.100510

[fsn33173-bib-0023] Jimoh, F. O. , Adedapo, A. A. , & Afolayan, A. J. (2010). Comparison of the nutritional value and biological activities of the acetone, methanol and water extracts of the leaves of *Solanum nigrum* and *Leonotis leonorus* . Food and Chemical Toxicology, 48(3), 964–971. 10.1016/j.fct.2010.01.007 20079394

[fsn33173-bib-0024] Kashfi, A. S. , Ramezan, Y. , & Khani, M. R. (2020). Simultaneous study of the antioxidant activity, microbial decontamination and color of dried peppermint (*Mentha piperita* L.) using low pressure cold plasma. LWT, 123, 109121. 10.1016/j.lwt.2020.109121

[fsn33173-bib-0025] Kha, T. C. , Nguyen, M. H. , & Roach, P. D. (2010). Effects of spray drying conditions on the physicochemical and antioxidant properties of the Gac (*Momordica cochinchinensis*) fruit aril powder. Journal of Food Engineering., 98, 385–392. 10.1016/j.jfoodeng.2010.01.016

[fsn33173-bib-0026] Kim, E. H. J. , Chen, X. D. , & Pearce, D. (2005). Effect of surface composition on the flowability of industrial spray‐dried dairy powders. Colloids and Surfaces B: Biointerfaces, 46(3), 182–187. 10.1016/j.colsurfb.2005.11.005 16337780

[fsn33173-bib-0027] Li, Q. , Li, X. , & Zhao, C. (2020). Strategies to obtain encapsulation and controlled release of small hydrophilic molecules. Frontiers in Bioengineering and Biotechnology, 8, 437. 10.3389/fbioe.2020.00437 32478055 PMC7237580

[fsn33173-bib-0028] Lourenço, S. C. , Moldão‐Martins, M. , & Alves, V. D. (2019). Antioxidants of natural plant origins: From sources to food industry applications. Molecules, 24(22), 4132. 10.3390/molecules24224132 31731614 PMC6891691

[fsn33173-bib-0029] Mahendran, T. (2010). Physicochemical properties and sensory characteristics of dehydrated guava concentrate: Effect of drying method and maltodextrin concentration. Tropical Agriculture, 13, 48–54. 10.4038/tare.v13i2.3138

[fsn33173-bib-0030] Martínez‐Ballesta, M. , Gil‐Izquierdo, Á. , García‐Viguera, C. , & Domínguez‐Perles, R. (2018). Nanoparticles and controlled delivery for bioactive compounds: Outlining challenges for new “smart‐foods” for health. Food, 7(5), 1–30. 10.3390/foods7050072 PMC5977092 29735897

[fsn33173-bib-0031] Medina‐Torres, L. , Santiago‐Adame, R. , Calderas, F. , Gallegos‐Infante, J. A. , González‐Laredo, R. F. , Rocha‐Guzmán, N. E. , & Manero, O. (2016). Microencapsulation by spray drying of laurel infusions (*Litsea glaucescens*) with maltodextrin. Industrial Crops and Products, 90, 1–8. 10.1016/j.indcrop.2016.06.009

[fsn33173-bib-0032] Messaoudi, R. , Abbeddou, S. , Mansouri, A. , Calokerinos, A. C. , & Kefalas, P. (2013). Phenolic profile and antioxidant activity of date‐pits of seven Algerian date palm fruit varieties. International Journal of Food Properties, 16(5), 1037–1047. 10.1080/10942912.2011.576355

[fsn33173-bib-0033] Mohammadi, M. , Soltani, M. , Siahpoosh, A. , & Mehrjan, M. S. (2016). Effects of date palm (*Phoenix dactylifera*) seed extract on heavy metals concentrations in carp (*Cyprinus carpio*). Polish Journal of Environmental Studies, 25(3), 1117–1123. 10.15244/pjoes/61853

[fsn33173-bib-0034] Moradi, D. , Ramezan, Y. , Eskandari, S. , Mirsaeedghazi, H. , & Dakheli, M. J. (2022). Optimization of polyphenol recovery from potato peel and its incorporation into low‐density polyethylene films activated by cold plasma. Biomass Conversion and Biorefinery, 1–15. 10.1007/s13399-022-03492-z

[fsn33173-bib-0035] Moser, P. , Telis, N. , Neves, N. A. , García‐Romero, E. , Gómez‐Alonso, S. , & Hermosín‐Gutiérrez, I. (2017). Storage stability of phenolic compounds in powdered BRS Violeta grape juice microencapsulated with protein and maltodextrin blends. Food Chemistry, 214, 308–318. 10.1016/j.foodchem.2016.07.081 27507480

[fsn33173-bib-0036] Najafi, M. N. , Kadkhodaee, R. , & Mortazavi, S. A. (2011). Effect of drying process and wall material on the properties of encapsulated cardamom oil. Food Biophysics, 6(1), 68–76.

[fsn33173-bib-0037] Negi, P. S. (2012). Plant extracts for the control of bacterial growth: Efficacy, stability and safety issues for food application. International Journal of Food Microbiology, 156(1), 7–17. 10.1016/j.ijfoodmicro.2012.03.006 22459761

[fsn33173-bib-0038] Oberoi, D. P. S. , & Sogi, D. S. (2015). Effect of drying methods and maltodextrin concentration on pigment content of watermelon juice powder. Journal of Food Engineering, 165, 172–178. 10.1016/j.jfoodeng.2015.06.024

[fsn33173-bib-0039] Oladzad, S. , Fallah, N. , Mahboubi, A. , Afsham, N. , & Taherzadeh, M. J. (2021). Date fruit processing waste and approaches to its valorization: A review. Bioresource Technology, 340, 125625. 10.1016/j.biortech.2021.125625 34332444

[fsn33173-bib-0040] Ourradi, H. , Ennahli, S. , Martos, M. V. , Hernadez, F. , Dilorenzo, C. , Hssaini, L. , & Hanine, H. (2021). Proximate composition of polyphenolic, phytochemical, antioxidant activity content and lipid profiles of date palm seeds oils (*Phoenix dactylifera* L.). Journal of Agriculture and Food Research, 6, 100217. 10.1016/j.jafr.2021.100217

[fsn33173-bib-0041] Peighambardoust, S. H. , & Sarabandi, K. (2017). Effect of spray drying conditions on physicochemical, functional properties and production yield of malt extract powder. Journal of Food Research, 27(2), 75–90. [In Persian].

[fsn33173-bib-0042] Pompeu, D. R. , Silva, E. M. , & Rogez, H. (2009). Optimisation of the solvent extraction of phenolic antioxidants from fruits of *Euterpe oleracea* using response surface methodology. Bioresource Technology, 100(23), 6076–6082. 10.1016/j.biortech.2009.03.083 19581082

[fsn33173-bib-0043] Poomkokrak, J. , Niamnuy, C. , Choicharoen, K. , & Devahastin, S. (2015). Encapsulation of soybean extract using spray drying. Journal of Food Science and Agricultural Technology, 1, 105–110.

[fsn33173-bib-0044] Quek, S. Y. , Chok, N. K. , & Swedlund, P. (2007). The physicochemical properties of spray‐dried watermelon powders. Chemical Engineering and Processing, 46, 386–392. 10.1016/j.cep.2006.06.020

[fsn33173-bib-0045] Santhalakshmy, S. , Bosco, S. J. D. , Francis, S. , & Sabeena, M. (2015). Effect of inlet temperature on physicochemical properties of spray‐dried jamun fruit juice powder. Powder Technology, 274, 37–43. 10.1016/j.powtec.2015.01.016

[fsn33173-bib-0046] Sarabandi, K. , & Sadeghi Mahoonak, A. (2016). The effect of inlet air temperature and the amounts of maltodextrin on physicochemical properties of spray dried date palm syrup. Innovative Food Technology, 4(2), 1–15. [In Persian]. 10.22104/JIFT.2016.338

[fsn33173-bib-0047] Šavikin, K. , Nastić, N. , Janković, T. , Bigović, D. , Miličević, B. , Vidović, S. , & Vladić, J. (2021). Effect of type and concentration of carrier material on the encapsulation of pomegranate peel using spray drying method. Food, 10(9), 1968. 10.3390/foods10091968 PMC8468628 34574078

[fsn33173-bib-0048] Selahvarzi, A. , Ramezan, Y. , Sanjabi, M. R. , Namdar, B. , Akbarmivehie, M. , Mirsaeedghazi, H. , & Azarikia, F. (2022). Optimization of ultrasonic‐assisted extraction of phenolic compounds from pomegranate and orange peels and their antioxidant activity in a functional drink. Food Bioscience, 49, 101918. 10.1016/j.fbio.2022.101918

[fsn33173-bib-0049] Shahidi, M. , & Molaveasi, M. (2020). Microencapsulation of cardamom essential oil with gum arabic, maltodextrin and inulin and the investigation of their physical‐chemical properties. Innovative Food Technology, 7(3), 433–446. [In Persian]. 10.22104/JIFT.2019.3813.1903

[fsn33173-bib-0050] Tolun, A. , Altintas, Z. , & Artik, N. (2016). Microencapsulation of grape polyphenols using maltodextrin and gum arabic as two alternative coating materials: Development and characterization. Journal of Biotechnology, 239, 23–33. 10.1016/j.jbiotec.2016.10.001 27720817

[fsn33173-bib-0051] Wagner, L. A. , & Warthesen, J. J. (1995). Stability of spray dried encapsulated carrot carotenes. Journal of Food Sciences, 60(5), 1048–1053. 10.1111/j.1365-2621.1995.tb06290.x

[fsn33173-bib-0052] Walton, D. E. (2000). The morphology of spray dried particles a qualitative view. Drying Technology, 18, 1943–1986. 10.1080/07373930008917822

[fsn33173-bib-0053] Wissam, Z. , Ghada, B. , Wassim, A. , & Warid, K. (2012). Effective extraction of polyphenols and proanthocyanidins from pomegranate's peel. International Journal of Pharmacy and Pharmaceutical Sciences, 4, 675–682.

[fsn33173-bib-0054] Zendeboodi, F. , Yeganehzad, S. , & Sadeghian, A. R. (2018). Production of carbohydrate‐protein based soft drink powder containing date syrup by spray dryer: Evaluation effect of drying carriers on physical properties of the powdered drink. Journal of Food Science and Technology, 15(78), 43–54. [In Persian].

